# Comparative Kinematic Analysis of Hurdle Clearance Technique in Dogs: A Preliminary Report

**DOI:** 10.3390/ani10122405

**Published:** 2020-12-16

**Authors:** Francisco Miró, Patricia López, Jose Manuel Vilar, Alfonso M. Galisteo, Joaquín Vivo, Juan L. Garrido-Castro, Luna Gutierrez-Cepeda

**Affiliations:** 1 Department Comparative Anatomy and Pathology, University of Córdoba, 14071 Córdoba, Spain; an1mirof@uco.es (F.M.); plsanchez@ucm.es (P.L.); an1magaa@uco.es (A.M.G.); an1viroj@uco.es (J.V.); 2 Veterinary Teaching Hospital, University of Las Palmas de Gran Canaria, Trasmontaña S/N, 35416 Arucas, Spain; 3 Maimonides Biomedical Research Institute (IMIBIC), 1404 Córdoba, Spain; juanluisgarrido@yahoo.es; 4 Animal Medicine and Surgery Department, Universidad Complutense de Madrid, 28040 Madrid, Spain; luna.gutierrez@vet.ucm.es

**Keywords:** dog, jumping, biomechanics, agility

## Abstract

**Simple Summary:**

Hurdle jumping is part of the increasingly popular canine agility competition. Although the jumping characteristics of agility dogs have been examined in recent years, there is currently a lack of data related to the suspension phase. The purpose of the present study was to investigate the biomechanics of the suspension phase of the agility jump and to analyze the kinematic differences in dogs with different jumping abilities. Two groups of dogs competing at different skill levels and assessed as excellent jumpers and less-skilled jumpers, respectively, were analyzed and compared. Excellent jumpers showed longer and faster jumps with flatter jump trajectories than less-skilled jumpers. In less-skilled jumpers, the distance in front of the hurdle was notably greater than the distance behind it, while the difference between these two distances was less in excellent jumpers. Length and duration of the jump, maximal height of the jumping trajectory, take-off and landing distances to the hurdle, time of occurrence of maximal jump height, and time of change in back orientation essentially defines the suspension phase of the agility jump. This study presents preliminary evidence that the kinematic characteristics of hurdle clearance are different in excellent jumper dogs and in less-skilled jumper dogs.

**Abstract:**

Although the jumping characteristics of agility dogs have been examined in recent years, there is currently a lack of data related to the suspension phase. The purpose of the present study was to investigate the biomechanics of the suspension phase of the agility jump and to analyze the kinematic differences in dogs with different jumping abilities. Two groups of dogs of the same height category (large dogs) competing at different skill levels and assessed as excellent jumpers (*n* = 4) and less-skilled jumpers (*n* = 3), respectively, were analyzed and statistically compared. Excellent jumpers showed longer and faster jumps with flatter jump trajectories than less-skilled jumpers. In less-skilled jumpers, the distance in front of the hurdle was notably greater than the distance behind it, while the difference between these two distances was less in excellent jumpers. Length and duration of the jump, maximal height of the jumping trajectory, take-off and landing distances to the hurdle, time of occurrence of maximal jump height, and time of change in back orientation essentially defines the suspension phase of the agility jump. This study presents preliminary evidence that the kinematic characteristics of hurdle clearance are different in excellent jumper dogs and in less-skilled jumper dogs.

## 1. Introduction

Dog agility is an increasingly popular sport discipline worldwide. In agility competitions, dogs need to negotiate different obstacles in a set order, at high speed, with the least possible faults. Many of the obstacles are hurdles, set at a predetermined height in relation to the dog’s height, according to the regulations of the Fédération Cynologique Internationale (FCI) [1].

The kinematics of hurdle clearance has been considered extensively in human athletes [2,3]. In quadrupeds, the kinematics of hurdle clearance has been analyzed in horses [4,5,6,7,8] and, more recently, in dogs [9,10,11,12,13,14,15]. In horses, the performance characteristics of good jumpers have been analyzed [4], and some contributing factors to successfully jump over different obstacles were determined [4,5,7,8]. These studies reveal that take-off and landing positions, the height of the center of gravity at the apex of the jump, and the duration of the suspension phase are among the most important biomechanical variables to investigate. Similarly, in agility dogs, some studies illustrate how jump kinematics become altered when the distance between hurdles is modified [11,12] or when the hurdle height increases [13,14]. It has been shown that, when jumping consecutive hurdles, more-skilled dogs take off and land farther away from the hurdle at a greater speed when compared to less-skilled dogs [12]. Another study [15] assessed how the jumping style of individual dogs, referred to as the topline angle during the bascule phase of the jump, affects jumping performance. This study concluded that larger topline angles during the bascule phase of the jump were associated with a reduced speed and that the jumping style does vary between height categories and breeds. Associations between injury characteristics and perceived causes of injury among dogs participating in agility-related activities have also been examined [16,17,18,19]. Different variables have been analyzed in these studies; however, research is needed to analyze the variables that best describe the kinematics of the suspension phase of the canine jump. We hypothesized that the kinematic characteristics of hurdle clearance would be different in excellent jumper dogs than in less-skilled jumper dogs.

One purpose of the present study was to investigate the kinematics of agility dogs during the suspension phase of the jump over the official corresponding hurdle. Another purpose was to identify the kinematic differences between dogs with different jumping abilities.

## 2. Materials and Methods

### 2.1. Subjects

The subjects of the study were seven agility dogs free of any pain or lameness according to a previous orthopedic and physical examination conducted by veterinarians. All seven dogs were competing in agility trials in the category of large dogs (43 cm or more at the withers—FCI, [1]). They were classified by the level within which they were currently participating and, additionally, into levels of skill by their ability jumping a 60-cm hurdle, assessed blindly by two highly experienced agility trainers. Long, low, and fast jumps were positively assessed by the trainers. Four of the dogs (numbers 1, 2, 3, and 4, Table 1), ranked by the trainers as excellent jumpers and currently competing at the highest level (level 3), were included in group A. Three dogs (numbers 5, 6, and 7, Table 1), ranked by the trainers as less-skilled jumpers and currently competing at level 2, were included in group B.

### 2.2. Measurement Protocol and Data Analysis

**Figure 1:**
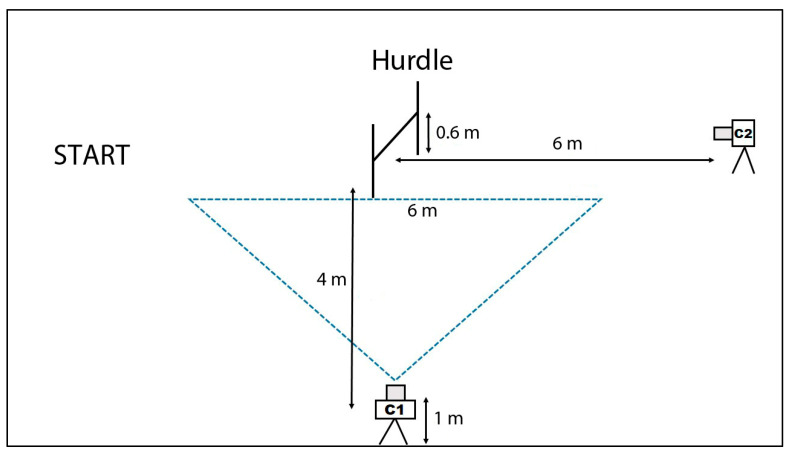
Schematic representation of the layout of the hurdle, cameras, and field of view of camera 1 used in the study. Dogs started to run from the start point towards the hurdle at their handler´s command. C1, camera for data collection. C2, camera for checking the validity of trials.

**Figure 2:**
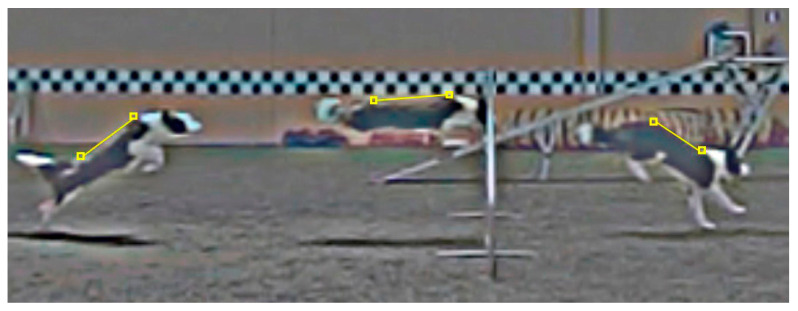
Virtual representation of take-off, maximal jump height, and landing sequences of an agility dog jumping over a 60-cm hurdle. Yellow marks and lines represent the withers, tuber sacrale, and back line.

All data were collected in one day at an indoor dog agility center equipped with artificial grass turf. After their regular warm-up protocol, each dog performed several jumps over a 60-cm single hurdle. Dogs stayed several meters in front of the hurdle, and they started to run towards the obstacle at their handler’s command. Two cameras (Casio Exilim^®^ EXZR100, Casio España, Barcelona, Spain) were used in the study. For data collection, one of the two cameras (C1, Figure 1) was placed perpendicular to the right of the dog’s line of motion and 4 m from the middle of the hurdle. The camera body was set on a tripod 1 m in height, and the zoom lens was adjusted to allow a 6-m field of view. The shutter speed was 240 fps. To assess the validity of each trial (the dog cleared the obstacle over the middle third of the hurdle, without touching the pole), another camera was aligned with the line of motion and placed 6 m away from the hurdle and in front of the fence. Five valid trials per dog were chosen for analysis. A 2D structure of known measurements was placed in the recording plane for 2D calibration purposes. Downstream data analysis was conducted using a UCOTrack^®^ (ISAB, Córdoba, Spain) video-based analysis system [20]. A low-pass filter (Butterworth, sampling frequency 480 Hz, cutoff frequency 15 Hz) was found to be more convenient at reducing the measurement noise that appeared during the analysis [21]. The suspension phase of the jump was determined between take-off and landing. Take-off was determined as the first frame with the trailing hind limb off the floor, and landing was determined as the first frame where the leading forelimb first contacted the ground (Figure 2). Based on previously published articles [11,12,15], the apparent locations of withers and tuber sacrale of the ilium (prominent point of the hip bone) were marked along the jump in the video images, and the back line was established between these two references. The back inclination angle, between the horizontal plane and the back line, was calculated throughout the suspension phase of the jump. The jump height, or the distance between the withers and the turf level, was also calculated throughout the entire suspension phase of the jump. Linear and angular measurements were automatically calculated by the system based on the calibrated coordinate system. Temporal measurements were calculated based on the number of frames and the shutter speed.

### 2.3. Jump Variables Assessed

The following temporal, linear, and angular variables were obtained, and results were expressed in tables in the Results section:

*Jump duration (dur):* time elapsing between take-off and landing.

*Percentage of take-off duration (% take-off dur):* duration (expressed as a percentage of the jump duration) from take-off to the instant in which the withers is exactly above the hurdle.

*Percentage of landing duration (% landing dur):* duration (expressed as a percentage of the jump duration) from the instant in which the withers is exactly above the hurdle to landing.

*Jump distance (jump dist):* horizontal distance between the withers at take-off and at landing.

*Percentage of take-off distance (% take-off dist):* horizontal distance between the withers at take-off and the vertical line at the hurdle, expressed as a percentage of the jump distance.

*Percentage of landing distance (% landing dist):* horizontal distance between the vertical line at the hurdle and the withers at landing, expressed as a percentage of the jump distance.

*Maximum jump height (max height):* maximum value of the jump height along the jump.

*Percentage of jump duration to maximum jump height (% max height dur):* time from take-off to the instant in which the withers reaches its maximum height, expressed as a percentage of the jump duration.

*Jump height at the hurdle (height at hurdle):* value of the jump height when the withers is exactly above the hurdle.

Difference between maximum jump height and jump height at the hurdle (max height-h. at h.).

Back inclination angle at take-off (back take-off).

Back inclination angle at landing (back landing).

*Range of motion of back inclination angle (back ROM):* difference between back inclination angle at take-off and at landing.

*Percentage of duration to horizontal back (% dur to Back O):* time from take-off to the instant at which the value of the back inclination angle is closest to zero, expressed as a percentage of the jump duration.

*Back over the hurdle (back at hurdle):* value of the back inclination angle when the withers is exactly above the hurdle.

**Figure 3:**
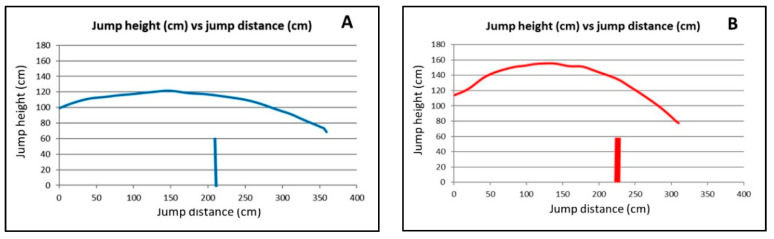
Graphic representation of the jump height (cm, *y*-axis) vs. jump distance travelled (cm, *x*-axis) of two agility dogs of different jumping abilities and competing at different levels. (**A**) dog number 1 of A group (excellent jumpers, competing in third and greater level of competition). (**B**) dog number 5, ranked as the worst jumper among all the dogs in group B (less-skilled jumpers, competing in second and lesser level of competition). Vertical line represents the 60-cm hurdle.

**Figure 4:**
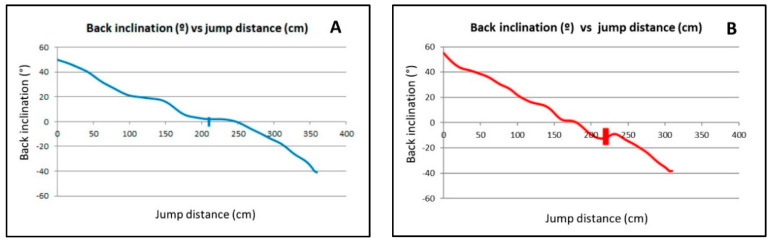
Graphic representation of the back inclination angle (°, *x*-axis) vs. jump distance travelled (cm *y*-axis) of two agility dogs jumping over a 60-cm high hurdle. (**A**) dog number 1 in group A (excellent jumpers, competing in third and greater level of competition). (**B**) dog number 5, ranked as the worst jumper among all the dogs of group B (less-skilled jumper, competing in second and lower level of competition). Vertical line represents the position of the hurdle in relation to the distance travelled.

To better understand the behavior of some variables, values of jump height versus jump distance travelled and values of back inclination angle versus jump distance travelled were expressed visually in Figure 3 and Figure 4, respectively, for one of the jumps from dog number 1 (excellent jumper, group A) and from dog number 5 (worst assessed jumper, group B).

### 2.4. Statistics

For each of the above variables, mean and standard deviation were calculated for each dog and group. A Mann–Whitney U test was used to assess any differences between groups. A normality test was completed (Shapiro–Wilk) in which almost all variables were not normally distributed due to the small sample size. Hence, non-parametric tests were performed. Values of *p* < 0.05 were considered significant.

## 3. Results

Results of the temporal and linear variables are shown in Table 2. There were significant differences (*p* < 0.05) between groups of dogs in percentage values of take-off duration, landing duration, take-off distance, and landing distance. The dogs in group B showed notably greater percentage values of take-off durations and distances than their corresponding landing values. In dogs in group A, the jump distances and durations in front of and behind the hurdle were not so different as they were in the dogs in group B. The jump distances were greater and the jump durations shorter in dogs in group A than in dogs in group B. The maximum jump height was lower in dogs in group A than in dogs in group B. In addition, in dogs in group A, there was no great difference between the maximum jump height values (max height, Table 2) and the corresponding values exactly above the hurdle (height at hurdle, Table 2). In dogs in group B, this difference was greater, although not significant (*p* < 0.05).

Results related to the back inclination angle are shown in Table 3. When the dogs were exactly above the hurdle, their backs were clearly tilted down in the dogs in group B, while their backs tended to be slightly tilted up in the dogs in group A. The percentages of duration to reach horizontal back (% dur to back 0, Table 3) were slightly greater than the corresponding values to reach the hurdle (% take-off dur, Table 2) in the dogs in group A. In dogs in group B, the percentages of duration to reach the horizontal back were smaller than the corresponding values to reach the hurdle.

## 4. Discussion

The study of sport dog movements, particularly jumping in agility dogs, is increasingly developing. Improved knowledge in the field could predict actual dog agility performance and improve techniques in training sessions.

The dogs used in the present study were previously observed by two expert agility trainers, and they were then ranked in two groups according to their jumping abilities. However, during a subjective evaluation, only a few kinematic variables can be perceived at a time. The evolution of computer-assisted analysis has improved the ability to quantitatively define temporospatial gait characteristics [9]. A validated motion capture and analysis system, SOMCAM3D^®^ (ISAB, Córdoba, Spain) [20], was used in the present study. Several similar studies have used markers glued on the dog’s skin as anatomical references [10,14]. In order to create greater feasibility and a simpler recording procedure as found in similar studies [11,12,13,15], we did not use markers. However, the anatomical references used in the present study could be easily located in the video images, as they were in the cited studies.

The horse’s jump has been broken into five parts: approach, take-off, suspension, landing, and departure [6]. The same phases and general characteristics can be described in the dog’s jump. To the authors’ knowledge, there is no complete description of the kinematics of the suspension phase in the jump of the agility dog. In addition, like in horses and humans, the individual characteristics of a dog jumping over a hurdle may be indeed detectable by using objective methods.

The aim of canine agility competitions is to negotiate different obstacles in a set order, within a fixed standard of time, and with the least faults. Faults are incurred for failure to negotiate the obstacles correctly and for failure to complete the course within the fixed standard of time [1]. An agility competition mixes both general motor abilities (speed, dynamic strength, and anaerobic endurance) and specific motor skills (jumping and quick turns). In addition to other training skills, ability and speed are equally important. Consequently, to clear a single hurdle, long and quick jumps should be considered as positive. It has been shown that beginner dogs jumped slower than higher-skilled dogs, illustrating how speed may contribute to dogs advancing up a competitive level or, arguably, how speed will increase with skill [11]. In the present study, dogs in group A showed longer and quicker jumps than dogs in group B.

During the approach and take-off phases of the jump, an animal has to choose an appropriate combination of forward velocity and distance to the obstacle to successfully clear the obstacle during the suspension phase [10]. The take-off position is described by the angle of the trunk, the associated height of the center of gravity with respect to the ground, and by the distances of the hind limbs to the obstacle [7]. In human [2,3] and equine [5,6,8] athletes, one of the important elements of jump kinematics is the accuracy of the approach to the obstacle. A study in horses revealed that the best jumpers are better than the worst jumpers at estimating the distance of their center of gravity to the fence [8]. In show jumping horses, the flight duration depends on the horizontal and vertical velocities at take-off and the difference between the heights of the center of gravity at take-off and landing [5]. The same authors affirmed that the horizontal velocity at take-off determines the horizontal distance covered during the suspension phase. Success is based upon the animal’s ability to adequately position its body at take-off to foresee the kinematic variables necessary to perform a successful jump. Another study [12] determined that more skilled large dogs took off and landed further away from the hurdle, when compared to less-skilled dogs. According to these authors, the experienced dogs may be better at deciphering the optimum take-off point not only to successfully clear the fence, but also to lengthen the jump and continue running. Data in the present study showed significant differences between the two groups of dogs in percentage values of take-off and landing distances. In less-skilled jumpers, the distance in front of the hurdle was notably greater than the distance behind it, while the difference between these two distances was less in excellent jumpers.

In horses, it has also been observed that the animals undergo vertical and horizontal displacement during the suspension phase of the jump [6]. The duration and length of this aerial phase are closely related [5]. At take-off, the path of the center of gravity and the angular momentum of the body around the center of gravity are established, and these properties cannot be changed during the suspension phase until the horse makes contact with an external object [4], such as the obstacle or the ground. The horizontal velocity of the center of gravity of jumping horses at take-off is significantly greater at wide jumps than at vertical fences [7]. In the present study, a flatter and lower trajectory of the body along the jump and greater jump distances travelled may have been a consequence of a greater component of horizontal velocity in the dogs in group A. Some authors [10,14] have influenced the jumping trajectory of agility dogs by varying the hurdle’s height. Dogs, like horses, adapt their jump kinematics to the height of the hurdle. It may be supposed that, whenever the obstacle is cleared, the flatter the jump is, the more energy is spent in forward movement and the less energy is wasted in upward movement. The dogs in group A in the present study showed flatter jump trajectories, with lower maximum jump height than the dogs in group B. For agility dogs, jumping over single 60-cm hurdles with longer and flatter jumps, as was the case in the present study, may better satisfy the objectives of the competition. The dogs in group A obtained the maximum height of the jump (value of % max height dur 42.53 ± 3.43; Table 2) a little before they passed over the hurdle (value of % take-off dur 54.65 ± 3.55; Table 2), while the dogs in group B reached the maximum height of the jump (value of % max height dur 41.97 ± 4.22; Table 2) far before they cleared the hurdle (value of % take-off dur 64.27 ± 6,69; Table 2). From a theoretical point of view, there should be an optimum displacement of the center of gravity during the suspension phase of the jump. Results of the present study showed the dog’s back clearly oriented upward from take-off to a specific point in the jump trajectory (% to back 0, Table 3); then, it rotates forward and downward to progressively reach more negative values in the back inclination angles until landing. The rate of rotation of the trunk during the suspension phase influences the horizontal velocity, which is clearly related to the length of the jump [6]. In the dogs in group A, the change of value from positive to negative (value of dur to back 0, 59.21 ± 5.31; Table 3) occurred shortly after clearing the hurdle (value of % take-off dur 54.65 ± 3.55; Table 2), while in the dogs in group B, this change (value of dur to back 0, 52.45 ± 5.50; Table 3) was quite earlier than the clearance of the hurdle (value of % take-off dur 64.27 ± 6.69; Table 2). When the dogs were exactly above the hurdle, the back was clearly tilted down in the dogs in group B, while backs tended to slightly tilt up in the dogs in group A. This may indicate a descending trajectory at the level of the hurdle in the dogs in group B, which may increase the chances of knockdowns in training and competition. Neck and shoulder muscle injuries are common in agility dogs due to consecutive forces experienced when landing from hurdles [17,18]. Hurdles are among the obstacles that put the shoulders of the agility dogs at risk [16]. The shoulder muscles are activated at high levels during jumping compared to walking and to other agility activities [19]. The dogs in group A of the present study cleared the hurdle with their back horizontal or slightly upwardly inclined, which may have ensured, once the hurdle was successfully cleared, to land far away from the hurdle. Although not exactly known, this functional aspect may contribute to the dogs in group A advancing to and maintaining level 3 in the competition.

Significant differences in jumping style between large and medium dogs and between collies and non-collies have been demonstrated [15]. In that study, jumping style was referred to as “the topline angle when the dog was exactly above the midpoint of the hurdle”. In our opinion, jumping style cannot be referred to as only the topline angle in one point of the trajectory. It is noteworthy that in the dogs in group A of the present study, the jump distance was greater and jump duration tended to be smaller than in the dogs in group B. In addition, in the dogs in group A, the jump distances and durations in front of and behind the hurdle were not so different, while the values of take-off durations and distances were notably greater than their corresponding landing values in the dogs in group B. All the above may support the suggestion that the dogs in group A were more efficient at adapting the pre-jump sprint to the outcome required. It has been cited [11] that skilled dogs appeared to be more adept at deciphering optimum kinematics than less-skilled dogs.

The lack of markers over anatomical references and the small sample size were the main limitations of the present study. Further studies with a greater number of dogs are necessary to verify the results and the kinematic differences observed between groups. However, we firmly consider that this study may be relevant in future research on agility dog jumping and may improve the understanding of kinematics in the suspension phase of the canine jump.

## 5. Conclusions

Based upon the results of the present study, it is concluded that the length and duration of the jump, maximal height of the jumping trajectory, take-off and landing distances to the hurdle, time of occurrence of the maximal jump height, and time of change in back orientation could essentially define the suspension phase of the agility jump. This study presents preliminary evidence that the kinematic characteristics of hurdle clearance are different in excellent jumper dogs and in less-skilled jumper dogs.

## Figures and Tables

**Table 1:** Details of agility dogs used in the study. Jump skill was assessed by two highly experienced agility trainers.

Dog	Breed	Age (years)	Height to the Withers (cm)	Weight (Kg)	Jump Skill Assessment (trainers)	Level of Competition
1	Border Collie	3.3	49	14	Excellent	3
2	Belgian M.	3	52	14.7	Excellent	3
3	Belgian M. mixed	4	55.1	18.3	Excellent	3
4	Border Collie	4	48	14	Excellent	3
5	Border Collie	7	50	15	Less-skilled	2
6	Mixed	4	57	27	Less-skilled	2
7	Bearded Collie	4	52	24	Less-skilled	2

Belgian M, Belgian Malinois Dog.

**Table 2:** Results (mean ± standard deviation) of temporal and linear variables of seven agility dogs jumping over a 60-cm hurdle. In bold results of Group A, dogs ranked as excellent jumpers competing in the greater level (level 3) and of Group B, dogs ranked as less-skilled jumpers and of a lower level in the competition (level 2). Jump distance was measured based on the withers position from take-off to landing. *, significant differences by a Mann–Whitney U test (*p* < 0.05).

Dog	1	2	3	4	Group A	5	6	7	Group B	
**dur (s)**	0.45 ± 0.01	0.45 ± 0.02	0.45 ± 0.02	0.45 ± 0.03	**0.45 ± 0.01**	0.60 ± 0.03	0.44 ± 0.03	0.44 ± 0.02	**0.49 ± 0.09**	
**% take-off dur**	58.40 ± 1.40	54.20 ± 3.90	50.00 ± 8.00	56.00 ± 7.00	**54.65 ± 3.55**	71.80 ± 1.20	59.00 ± 8.00	62.00 ± 2.00	64.27 ± 6.69 *	
**% landing dur**	41.60 ± 1.40	45.80 ± 3.90	50.00 ± 8.00	44.00 ± 7.00	**45.35 ± 3.74**	28.20 ± 1.20	41.00 ± 8.00	38.00 ± 0.02	35.73 ± 7.28 *	
**jump dist (m)**	3.50 ± 0.05	3.48 ± 0.31	3.91 ± 0.09	3.81 ± 0.27	**3.47 ± 0.37**	3.15 ± 0.11	3.19 ± 0.29	2.50 ± 0.14	**2.95 ± 0.39**	
**% take-off dist**	58.70 ± 1.60	52.20 ± 0.70	52.00 ± 8.00	58,9 ± 7.00	**55.23 ± 3.62**	73.00 ± 0.70	59.00 ± 8.00	63.00 ± 2.00	65.00 ± 7.21 *	
**% landing dist**	41.30 ± 1.60	47.70 ± 0.70	48.00 ± 8.00	41.1 ± 7.00	**44.50 ± 3.87**	27.00 ± 0.70	42.00 ± 8.00	37.00 ± 2.00	35.00 ± 7.21 *	
**max height (m)**	1.20 ± 0.01	1.22 ± 0.03	1.03 ± 0.04	1.01 ± 0.03	**1.12 ± 0.11**	1.56 ± 0.07	1.04 ± 0.04	1.14 ± 0.01	**1.25 ± 0.28**	
**% max height dur**	40.30 ± 1.00	39.20 ± 8.00	46.70 ± 4.16	43.98 ± 2.27	**42.53 ± 3.43**	45.50 ± 2.90	43.1 ± 6.14	37.3 ± 2.66	**41.97 ± 4.22**	
**height at hurdle (m)**	1.14 ± 2.40	1.20 ± 0.02	1.00 ± 0.05	0.99 ± 0.03	**1.08 ± 0.10**	1.33 ± 0.05	1.02 ± 0.05	1.09 ± 0.03	**1.15 ± 0.16**	
**max height-h. at h.(m)**	0.06 ± 0.02	0.02 ± 0.01	0.03 ± 0.04	0.02 ± 0.02	**0.03 ± 0.02**	0.23 ± 0.03	0.02 ± 0.02	0.05 ± 0.04	**0.10 ± 0.11**	

**Table 3:** Results (mean ± standard deviation) of back inclination angle of seven agility dogs jumping over a 60-cm hurdle. In bold results of Group A, dogs ranked as excellent jumpers and competing in the greater level (level 3) and of Group B, dogs ranked as less-skilled jumpers and of a lower level in the competition (level 2). *, significant differences by a Mann–Whitney U test (*p* < 0.05).

og	1	2	3	4	Group A	5	6	7	Group B	
**back take-off (°)**	47.68 ± 2.23	42.21 ± 1.40	31.00 ± 2.65	32.8 ± 2.69	**38.42 ± 7.91**	54.77 ± 2.61	27.7 ± 3.94	34.6 ± 2.05	**39.02 ± 14.07**	
**back landing (°)**	−38.96 ± 3.55	−35.38 ± 7.37	−36.12 ± 3.46	−38.6 ± 2.74	**−37.24 ± 1.81**	−36.46 ± 1.63	−37.45 ± 0.51	−36.65 ± 3.35	**−36.49 ± 0.50**	
**back ROM (°)**	86.64 ± 5.33	77.59 ± 6.09	67.23 ± 5.05	71.4 ± 4.02	**75.66 ± 8.51**	91.23 ± 3.09	64.64 ± 4.17	70.71 ± 4.42	**75.51 ± 13.95**	
**% dur to back**	63.19 ± 5.95	64.29 ± 6.83	53.81 ± 2.64	55.55 ± 5.63	**59.21 ± 5.31**	55.54 ± 2.09	46.13 ± 6.86	55.76 ± 3.03	**52.45 ± 5.50**	
**back at hurdle (°)**	1.28 ± 0.81	3.15 ± 1.82	2.55 ± 4.64	−0.32 ± 1.52	**1.67 ± 1.51**	−14.86 ± 3.92	−7.47 ± 7.06	−8.34 ± 4.87	−10.21 ± 4.05 *	
